# Celecoxib and sulfasalazine had negative association with coronary artery diseases in patients with ankylosing spondylitis: A nation-wide, population-based case-control study

**DOI:** 10.1097/MD.0000000000004792

**Published:** 2016-09-09

**Authors:** Li-Chih Wu, Pui-Ying Leong, Kai-Jieh Yeo, Ting-Yu Li, Yu-Hsun Wang, Jeng-Yuan Chiou, James Cheng-Chung Wei

**Affiliations:** a Institute of Medicine, Chung Shan Medical University; b Division of Allergy, Immunology and Rheumatology, Institute of Medicine, Chung Shan Medical University; c Department of Medical Research, Chung Shan Medical University Hospital; d Division of Allergy, Immunology and Rheumatology, Chung Shan Medical University Hospital; Institute of Medicine, Chung Shan Medical University; Institute of Integrative Medicine, China Medical University, Taichung, Taiwan, No.110, Sec.1, Jianguo N. Rd., Taichung City 40201, Taiwan; e School of Health Policy and Management, Chung Shan Medical University, Taichung, Taiwan.

**Keywords:** ankylosing spondylitis, celecoxib, coronary artery disease, defined daily dose, sulfasalazine

## Abstract

The aim of the study is to assess the effects of celecoxib and sulfasalazine on the risk of coronary artery disease (CAD) in patients with ankylosing spondylitis (AS).

Using the claims data of Taiwan National Health Insurance (NHI) database, a nationally representative data that contain the medical records of 23 million Taiwan residents, we randomly selected 1 million cohort from the database, and then we enrolled only patients who were newly diagnosed with AS (n = 4829) between year 2001 and 2010, excluding patients who had CAD (ICD-9- CM codes: 410–414) before the diagnosis of AS (n = 4112). According to propensity score matched 1:2 on age, gender, AS duration, Charlson comorbidity index, hypertension, and hyperlipidemia, 236 and 472 patients were included in the case (AS with CAD) and control (AS without CAD) groups, respectively. We used the WHO defined daily dose (DDD) as a tool to assess the dosage of sulfasalazine and celecoxib exposure. Conditional logistic regression was used to estimate the crude and adjusted odds ratios (ORs) and 95% confidence interval (CI) for the risk of CAD associated with use of sulfasalazine and celecoxib.

Among 4112 AS patients, 8.4% (346/4112) developed CAD. CAD in AS patients were positively associated with age of 35 to 65, Charlson comorbidities index (CCI), hypertension, and hyperlipidemia. There was no gender difference between case and control groups. After adjustment for age, gender, CCI, hypertension, and hyperlipidemia, sulfasalazine users with an average daily dose ≥ 0.5 DDD (0.5 gm/day) had negative association with CAD events as compared to sulfasalazine nonusers (OR 0.63; 95% CI, 0.40–0.99, *P* < 0.05). NSAIDs, including celecoxib, etoricoxib, but no naproxen and diclofenac were negatively associated with CAD. Celecoxib users, with an average daily dose > 1.5 DDD, were negatively associated with CAD events, compared to celecoxib nonusers (OR 0.34; 95% CI, 0.13–0.89; *P* < 0.05).

In this 10-year population-based case-control study, 8.4% of AS patients developed CAD. Sulfasalazine usage at an average dose of ≥ 0.5 gm/day demonstrated negative association with CAD events in patients with AS.

## 1 Introduction

Ankylosing spondylitis (AS) is a type of inflammatory arthritis involving the axial skeleton characterized by ankylosis and chronic back pain, which can lead to structural and functional impairments and a decline in quality of life. The usual age of onset is from the late teens to 40 years of age. Two times more frequent in men than in women.^[1]^ The standard treatment of spinal symptoms for patients with AS has consisted of nonsteroidal anti-inflammatory drugs (NSAIDs).^[2]^ NSAIDs are the most widely prescribed drugs for the treatment of acute and chronic pain, but they have gastrointestinal adverse effects.^[3]^ Cyclooxygenase-2 (COX II) inhibitors (for example, celecoxib and etoricoxib) were developed with the aim of reducing the incidence of serious GI adverse effects associated with the administration of traditional NSAIDs. Furthermore, the relation between COX II inhibitors and cardiovascular disease risk, including coronary artery diseases (CAD) has been mentioned in several studies.^[4,5]^ Disease-modifying antirheumatic drugs (DMARDs), such as sulfasalazine, are candidates for second-line treatment of AS when a patient becomes refractory to NSAIDs or has persistent articular involvement.^[2]^ Some studies have indicated that DMARDs may cause reduced risks of CAD in RA patients.^[6]^ But whether COX II inhibitors or DMARDs associated with a risk of CAD has rarely discussed in AS patients. Recent research suggests that cardiovascular event is the major cause of death in AS patients. Patients with AS need to be monitored to lower risk of CAD insofar as possible.^[7,8]^ Consequently, we want to know whether the use of NSAID and sulfasalazine in AS patients is related to the risk of CAD.

## 2 Methods

This study was approved by Institutional Review Board of Chung Shan Medical University Hospital, Taiwan. (CSMUH No: CS13021).

### 2.1 Data sources

The Taiwan National Health Insurance (NHI) Program has been implemented in Taiwan since 1995, and it has since covered ∼99% of the total 23 million people living in Taiwan.^[9]^ In this study, we used a subset of the National Health Insurance Research Database (NHIRD), the Longitudinal Health Insurance Database (LHID), which comprises the patient data from 2001 to 2010. The LHID includes the original claim data of 1,000,000 beneficiaries randomly sampled from the original NHIRD. The database includes encrypted personal information, such as demographic data, disease diagnosis, medications, and treatments. The diagnosis of disease is based on the International Classification of Diseases, Ninth Revision, Clinical Modification (ICD-9-CM codes).

### 2.2 Identification of cases and controls

**Figure 1:**
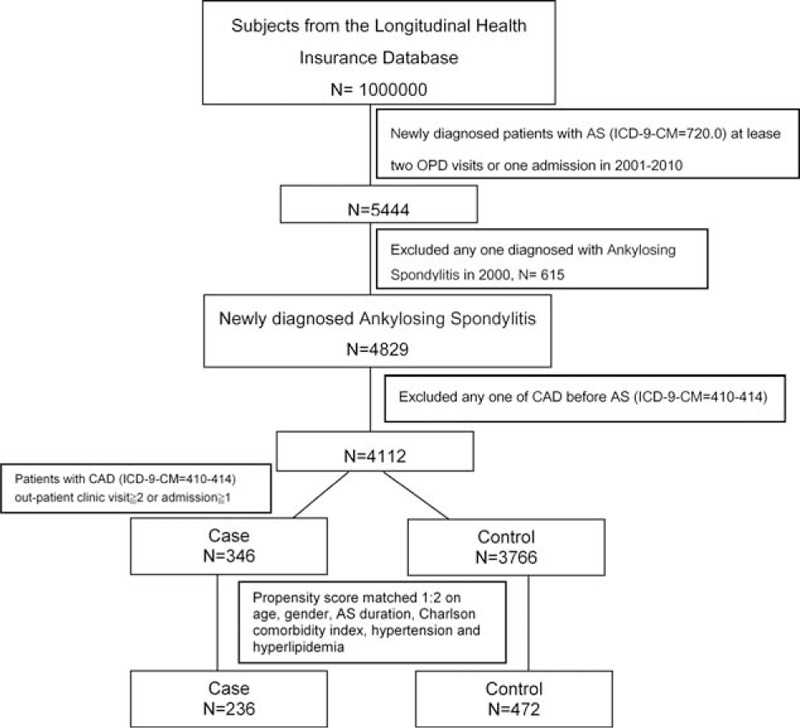
Consort diagram. AS = ankylosing spondylitis, CAD = coronary artery disease, LHID = longitudinal health insurance database.

We randomly selected 1 million patients from the database, and then we enrolled only patients who were newly diagnosed with AS (ICD-9-CM code: 720.0 and had outpatient department visit ≧2 or admission ≧1) between year 2001 and 2010 (n = 4829), excluding patients who had CAD (ICD-9- CM codes: 410–414) before the diagnosis of AS (n = 4112). Among these AS patients, 346 patients who were diagnosed with *CAD* (ICD-9-CM code: 410–414 and had outpatient department visit ≧2 or admission ≧1) were included as cases. The other 3766 AS patients without CAD served as the control group. Furthermore, because hypertension and hyperlipidemia were the risk factor of CAD, we also chose both of them to be the variables to matching. By propensity score matching 1:2 on age, gender, AS duration, Charlson comorbidity index, hypertension and hyperlipidemia, 236 and 472 patients were included in the case and control groups, respectively (Fig. 1).

### 2.3 Statistical analysis

We used the WHO defined daily dose (DDD) as a tool to assess the frequency of sulfasalazine and celecoxib exposure. The DDD is the average adult dose for a drug recommended for its main indication.^[10]^ The endpoint of CAD (ICD-9-CM codes 410–414) was used as outcome of sulfasalazine and celecoxib exposure.

Conditional logistic regression was used to estimate the crude and adjusted odds ratios (ORs) and 95% confidence interval (CI) for the risk of CAD associated with use of sulfasalazine and celecoxib. A 2-tailed *P* value <0.05 was considered significant. Potential risk factors including sex, age, CCI, AS disease duration hypertension (ICD-9-CM codes 401–405), hyperlipidemia (ICD-9-CM codes 272.0–272.4), and other drugs used such as etoricoxib, naproxen, and diclofenac were incorporated into the models. The statistical analyses in this study were executed by SPSS version 18.0.

## 3 Results

### 3.1 Characteristics of the study population

In Table 1, among 4112 AS patients, 8.4% (346/4112) developed CAD. CAD in AS patients were positively associated with age of 35 to 65, Charlson comorbidities index (CCI), hypertension, and hyperlipidemia. There was no gender difference between case and control groups. This study comprises 236 cases of subjects diagnosed with CAD in their AS duration and 472 controls not diagnosed with CAD in their AS duration, with similar distributions of sex, age, AS duration, Charlson comorbidity index, hypertension, and hyperlipidemia. The mean age (standard deviation) of the case group and the control group were 55.9 ± 14.3 years and 54.8 ± 14.8 years, respectively (*P* = 0.335). More than 70% of subjects were aged <65 years old. As shown, the case group had lower proportions of use of celecoxib (22.9% vs 30.5%, *P* = 0.033) and sulfasalazine (19.5% vs 29.2%, *P* = 0.005).

**Table 1:**
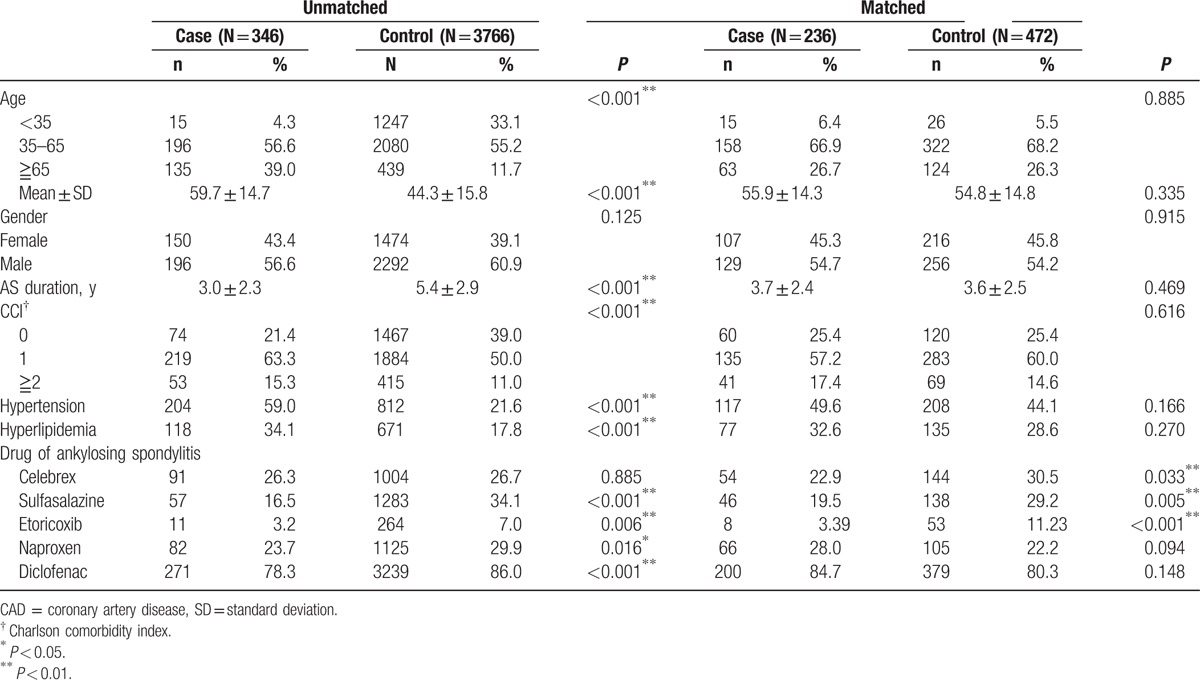
Demographic data and risk factors for CAD.

### 3.2 Odds ratio of CAD with celecoxib use and sulfasalazine use

**Figure 2:**
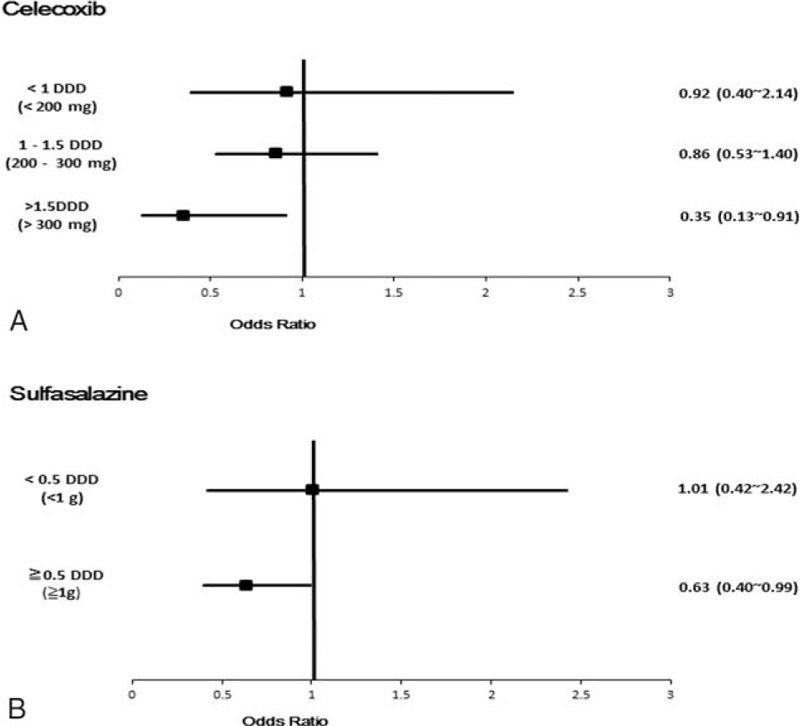
Risks of all types of coronary artery diseases associated with (A) celecoxib, (B) sulfasalazine. For celecoxib users, drug exposure was categorized into < 1 DDD (<200 mg), 1–1.5 DDD (200–300 mg), > 1.5 DDD (>300 mg); for sulfasalazine users, drug exposure was categorized into < 0.5 DDD (1 g), ≧0.5 DDD (≧ 1 g). DDD = defined daily dose.

Table 2 shows the crude and adjusted ORs of CAD associated with the use of sulfasalazine, celecoxib, and the presence of comorbidities. Compared with sulfasalazine nonusers, after adjusting for age, gender, Charlson comorbidity index, hypertension, and hyperlipidemia, the OR of CAD was 0.66 for use of sulfasalazine with significant difference (95% CI, 0.44–0.999, *P* < 0.05). Then, we further stratified sulfasalazine use into 2 groups by DDD (Fig. 2). We used 0.5DDD (1 g) as the cutoff point, categorizing subjects into the low-dose group and the high-dose group. Sulfasalazine users with an average daily dose ≥0.5 DDD (≥1 g) had negative association with CAD events as compared to sulfasalazine nonusers (OR 0.63; 95% CI, 0.40–0.99, *P* < 0.05).

**Table 2:**
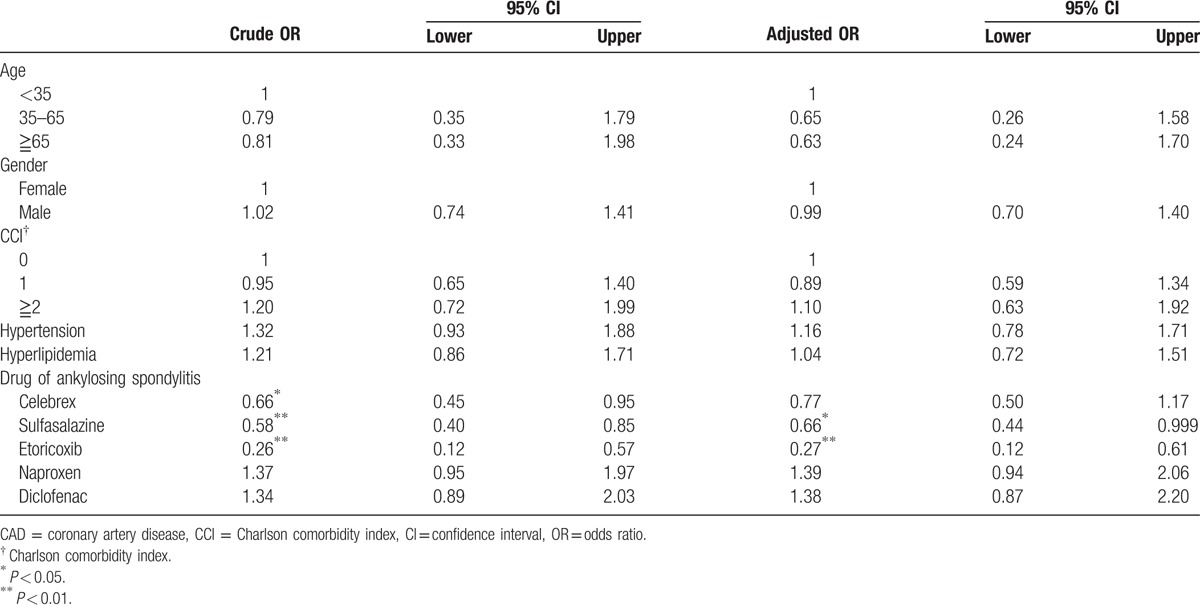
Risk factors and their odds ratios for CAD events.

NSAIDs, including celecoxib, etoricoxib, but no naproxen and diclofenac were negatively associated with CAD (Table 2). We further stratified celecoxib use into 3 groups by 1 DDD (200 mg) and 1.5 DDD (300 mg). The result was demonstrated in Fig. 2. Celecoxib users, with an average daily dose >1.5 DDD (>300 mg), were negatively associated with CAD events, compared to celecoxib nonusers (OR 0.34; 95% CI, 0.13 0.89; *P* < 0.05).

## 4 Discussion

Our study demonstrated that AS patients treated with celecoxib and sulfasalazine both did have a lower risk of CAD than that of nonusers. In high-dose users, there was a trend showing that the higher the dose was, the lower the risk was. AS patients with both an average daily dose >1.5 DDD celecoxib and ≥0.5 DDD sulfasalazine had reduced risk of CAD.

As the VIGOR study demonstrated that rofecoxib was associated with an increased risk of myocardial infarction,^[11]^ concern about the cardiovascular risk with COX2-selective drugs has been emerged recently. In the APC trial, it reported that patients treated with celecoxib 200 mg twice daily and with 400 mg twice daily had a 2 fivefold and 3 fourfold increase in the cardiovascular risk, respectively.^[12]^ In the APPROVE trial, it also proved that COX II inhibitors was associated with a dose-related risk of cardiovascular disease such as myocardial infarction, stroke, or heart failure.^[13]^ However, because these trials enrolled patients with colon adenoma, not a rheumatic diseases, it was not sure whether their results could also be generalized to suggest a cardiovascular adverse effect of celecoxib in patients with any autoimmune diseases.

In other studies, no evidence of increased risk for celecoxib was seen.^[14–16]^ Indeed, several studies suggested that celecoxib improved endothelial function and also had potentially beneficial effects on coronary artery blood flow.^[17,18]^ Besides, a case-control study indicated that celecoxib was more negatively associated with occurrence of nonfatal myocardial infarction compared with NSAIDs nonusers or rofecoxib users.^[19]^ Further evidence, especially randomized trials are still necessary to figure out that the impact of long-term use of celecoxib confers a protective effect or a risk of CAD for AS patients.

Recent studies suggest that DMARDs use, such as methotrexate, sulfasalazine, and leflunomide, is associated with a reduced risk of CAD in rheumatoid arthritis (RA) patients.^[6,20]^ However, it was questioned whether the result of these studies in RA patients can also be the same as that in AS patients. Evidence of increased cardiovascular risk in AS patients is unclear now. A likely explanation may be that DMARDs have an ability to reduce systemic inflammation and that can mediate a decrease in cardiac risk.^[6]^

This study had certain limitation. Because the incidence of CAD should be followed up in a long period, the risk of CAD associated with newly diagnosed AS in this study might be underestimated. In this study, ICD-9 codes (410–414) were chosen to be the diagnosis of CAD. However, database codes such as ICD-9 cannot defined the disease activity of each patients as accurately as angiography, treadmill, or nuclear medicine data did. Besides, though we have adjusted for as many confounders as we could, in this case-control study, bias due to unknown confounding factors might still remain. Furthermore, whether the results in this study can be generalized to AS patients in other countries or even patients with other diseases is still an issue to be discussed.

However, our study uses DDD to analyze the dosage of sulfasalazine and celecoxib with the risk of CAD in AS, more accurate than other studies to define how many doses of sulfasalazine and celecoxib have negative association with CAD events. For sulfasalazine and celecoxib are widely used in AS patients, to figure out whether both drugs can be safe options for AS patients and even benefits them is of great significance. Further researches providing concrete evidence of the safety of these 2 drugs are still needed.

## 5 Conclusion

In this 10-year population-based case-control study, 8.4% of AS patients developed CAD. Sulfasalazine usage at an average dose of ≥0.5 gm/day demonstrated negative association with CAD events in patients with AS.
